# A Novel Approach to Combating Antibiotic Resistance: A Chitosan-Based Nanocomposite with Green AgNPs and Gentamicin

**DOI:** 10.3390/ijms27021036

**Published:** 2026-01-20

**Authors:** Mukil Madhusudanan, Priyanka Singh, Viney Ghai, Santosh Pandit, Roland Kádár, Ivan Mijakovic

**Affiliations:** 1 The Novo Nordisk Foundation Center for Biosustainability, Technical University of Denmark, DK-2800 Kogens Lyngby, Denmark; 2 Department of Industrial and Materials Science, Chalmers University of Technology, 41296 Göteborg, Sweden; 3 Systems and Synthetic Biology Division, Department of Life Sciences, Chalmers University of Technology, 41296 Göteborg, Sweden

**Keywords:** green synthesis, chitosan nanocomposite, silver nanoparticles, gentamicin, antimicrobial resistance

## Abstract

This study investigates the synthesis of silver nanoparticles (AgNPs) using *Crassula ovata* (Jade plant) leaf extract and their subsequent incorporation into chitosan-based nanocomposite films for enhanced antimicrobial activity against four pathogenic microorganisms: *Escherichia coli*, *Pseudomonas aeruginosa*, *Staphylococcus epidermidis*, and Methicillin-resistant *Staphylococcus aureus*. Jade AgNPs were chosen for their ease of synthesis, stability, and potent antimicrobial activity. Chitosan encapsulation improved the stability of AgNPs and enhanced their interaction with bacterial cells, leading to improved bactericidal performance. The addition of gentamicin to the nanocomposite further amplified antibacterial activity, reducing the MBC values from 10 to 4 µg/mL for *E. coli*, 12.5 to 6 µg/mL for *P. aeruginosa*, 10 to 6 µg/mL for *S. epidermidis*, and 15 to 8 µg/mL for MRSA, compared to AgNPs alone. Mechanical characterization using dynamic mechanical analysis revealed improved robustness, with storage modulus increasing from approximately 24 MPa for chitosan-AgNPs films to 36 MPa for gentamicin-loaded nanocomposite films, while maintaining elasticity. Overall, these multifunctional nanocomposite films demonstrate strong antimicrobial activity and improved mechanical performance, supporting further evaluation as candidate materials for wound-related antimicrobial applications and localized infection control strategies. Such localized antimicrobial platforms may also contribute to strategies aimed at mitigating antibiotic resistance.

## 1. Introduction

Antimicrobial resistance (AMR) is a rapidly escalating global health concern, driven by the excessive use of antibiotics and the increasing prevalence of multidrug-resistant pathogens [1,2,3]. In clinical settings, persistent infections caused by resistant strains remain particularly challenging, often requiring prolonged treatment and high antibiotic dosages [4]. This situation underscores the urgent need for novel antimicrobial systems that are effective, sustainable, and capable of reducing selective pressure associated with conventional antibiotic therapies [5]. In this context, nanomaterials have emerged as valuable alternatives, owing to their tunable physicochemical properties and their ability to target bacteria through multiple mechanisms, thereby lowering the likelihood of resistance development [6].

Among the various nanomaterials investigated, silver nanoparticles (AgNPs) have gained extensive attention for their broad-spectrum antimicrobial properties and multifaceted mechanisms of action, which include disruption of cell membranes, generation of reactive oxygen species, and interference with essential metabolic pathways [7,8]. However, conventional methods for synthesizing AgNPs often rely on toxic reducing agents and energy-intensive processes, limiting their safety and applicability in biomedical settings [9]. Green synthesis of nanoparticles using plant extracts provides a more sustainable alternative, offering a simple, environmentally friendly approach that utilizes phytochemicals as natural reducing and stabilizing agents [10]. *Crassula ovata* (Jade plant), a succulent abundant in phenolic compounds and flavonoids, is particularly suitable for this purpose [11]. Its phytochemicals provide an effective matrix that both reduces silver ions and stabilizes the resulting nanoparticles, producing uniform AgNPs with strong antimicrobial potential that have been widely explored for biomedical applications.

While plant-mediated synthesis of AgNPs has been widely explored, *Crassula ovata* offers a robust and accessible phytochemical source that enables rapid nanoparticle formation and surface capping through polyphenols and other reducing agents, potentially improving dispersion stability and compatibility with polymer matrices. In our recent comparative study of green and chemically synthesized AgNPs, *C. ovata*-derived AgNPs showed enhanced antimicrobial potency against clinically relevant bacterial pathogens, supporting their selection for incorporation into chitosan-based films [12,13,14,15].

To enhance the performance, stability, and applicability of green-synthesized AgNPs, biopolymers such as chitosan have been widely explored [16,17,18]. Chitosan is a linear polysaccharide derived from the deacetylation of chitin, a naturally occurring polymer found in crustacean exoskeletons [19]. It is recognized for its biocompatibility, biodegradability, and intrinsic antimicrobial activity [20]. Its positively charged amino groups interact electrostatically with negatively charged bacterial membranes, resulting in membrane destabilization and promoting the uptake of AgNPs into bacterial cells, thereby improving the overall efficacy of the composite [21,22]. Furthermore, chitosan readily forms nanoparticles and flexible films, enabling the development of advanced materials for antimicrobial, drug delivery, and wound-related applications [20,23]. Incorporating AgNPs into a chitosan matrix creates a stable, protective layer around the nanoparticles that can increase their longevity and environmental stability, while also enabling enhanced antimicrobial effects arising from the combined action of both materials [24,25].

Beyond polymer encapsulation, coupling AgNPs with conventional antibiotics represents a powerful approach to further strengthening antimicrobial performance [26,27]. Gentamicin, a broad-spectrum aminoglycoside antibiotic, is frequently used to treat infections caused by Gram-negative bacteria and several Gram-positive species [28]. It exerts its antibacterial activity by binding to the 30S ribosomal subunit, disrupting protein synthesis, and leading to cell death [29]. Integrating gentamicin with AgNPs and chitosan offers the potential for a multifunctional antimicrobial system in which each component contributes complementary mechanisms of action. Such combined action can enhance antibacterial potency, reduce the required antibiotic dose, and minimize the emergence of resistant strains [26,27,30]. The loading of gentamicin into chitosan-AgNPs systems is also advantageous for applications requiring localized drug delivery in topical antimicrobial settings, including wound-associated infections.

The development of multifunctional composite materials that combine green-synthesized AgNPs, biopolymeric carriers, and conventional antibiotics remains an area of active research [10,31,32]. While the antimicrobial properties of each component have been individually explored, there are relatively few studies examining their integration into a unified nanocomposite system with both strong antibacterial performance and functional mechanical properties. Such materials could potentially offer significant benefits for wound management, where prolonged antimicrobial action, moisture retention, biocompatibility, and structural integrity are essential. Chitosan-based films incorporating AgNPs and antibiotics have shown promise, but further investigation is needed to fully understand how these components interact and how their combined properties can be optimized for biomedical applications. Recent reviews have highlighted the importance of combining antimicrobial function with mechanical robustness and controlled diffusion behaviour in wound dressing materials [33].

For wound healing applications, mechanical properties play a crucial role in determining the durability, flexibility, and overall performance of polymer films [34]. Wound dressings must maintain sufficient stiffness and structural integrity to protect the wound site, while also exhibiting adequate elasticity to withstand deformation caused by body movement without cracking or delamination [35]. Materials with a high storage modulus (G′) and a low loss tangent (tan δ) are often preferred, as these characteristics indicate greater elastic response, reduced energy dissipation, and strong resistance to mechanical stress. Incorporating nanoparticles or bioactive molecules into chitosan-based films can therefore influence not only antimicrobial functionality but also the mechanical robustness of the final composite. Establishing whether the mechanical properties of the synthesized films meet these requirements is essential for evaluating their potential for biomedical applications such as wound dressings.

Therefore, the present study focuses on developing a multifunctional chitosan-based nanocomposite incorporating green-synthesized Jade AgNPs and gentamicin, with the aim of enhancing antimicrobial activity, stability, and potential relevance for wound-related applications. By leveraging the complementary properties of chitosan, AgNPs, and gentamicin, this work seeks to create a composite material capable of potentially providing sustained, localized antimicrobial action while maintaining favourable mechanical characteristics. The physicochemical properties, stability, and antibacterial efficacy of these nanocomposites were systematically evaluated against clinically relevant pathogens, offering important insights into their potential for future biomedical use.

## 2. Results

### 2.1. Encapsulation of AgNPs Increases Particle Size and Surface Charge

**Figure 1:**
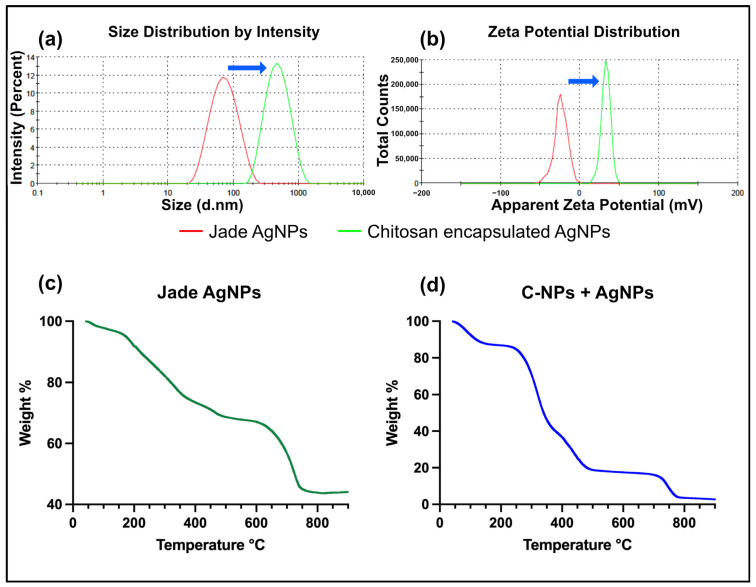
DLS measurements (**a**) Hydrodynamic diameter of Jade AgNPs (red) and chitosan-coated AgNPs (green), (**b**) Zeta potential of Jade AgNPs (red) and chitosan-coated Jade AgNPs (green). TGA spectra of (**c**) Jade AgNPs, and (**d**) Chitosan-coated AgNPs.

The slight turbidity of the reaction mixture confirmed the formation of C-NPs. DLS analysis was performed to assess the hydrodynamic diameter and size distribution of the nanoparticles. The Jade AgNPs had a Z-average of 62.5 nm and a PDI of 0.197, signifying a relatively uniform particle size distribution [36]. When C-NPs were combined with AgNPs, a noticeable increase in particle size was observed, with an average diameter of 402.2 nm and a PDI of 0.212, suggesting a moderately uniform size distribution of the polymer-coated AgNPs. The absence of minor peaks suggests the effective encapsulation of AgNPs into C-NPs (Figure 1a).

The zeta potential analysis of the Jade AgNPs revealed a surface charge of −23.7 mV, indicating moderate colloidal stability due to electrostatic repulsion between the particles. However, after encapsulation with chitosan, the zeta potential shifted significantly to +33 mV, reflecting a strong positive charge (Figure 1b). This shift suggests improved stability, as the high positive zeta potential enhances electrostatic repulsion, preventing agglomeration and providing better dispersion in colloidal systems. Together, the observed Z-average size distribution and the measured zeta potential suggest a stable colloidal dispersion under the tested conditions, supporting the effectiveness of plant-derived capping and chitosan encapsulation in limiting aggregation. The change in zeta potential following chitosan encapsulation, therefore, supports enhanced dispersion stability and improved nanoparticle performance for further evaluation in antimicrobial biomaterial applications.

### 2.2. Chitosan Encapsulation Alters Thermal Degradation Profile

The thermal degradation analysis of Jade AgNPs and chitosan-encapsulated AgNPs shows distinct differences. For plant extract-synthesized AgNPs, the degradation begins with a gradual weight loss, likely from moisture and volatile organic compounds, followed by a significant drop at higher temperatures (200–300 °C), indicating the decomposition of organic materials or plant-derived stabilizing agents around the AgNPs (Figure 1c). In contrast, the chitosan-encapsulated AgNPs exhibit a multi-stage degradation process (Figure 1d). The initial weight loss below 100 °C is attributed to moisture, followed by substantial degradation between 200 °C and 400 °C due to the thermal decomposition of the chitosan polymer matrix. The thermal degradation between 600 °C and 800 °C in both Jade AgNPs and chitosan-encapsulated AgNPs likely results from the combustion of remaining carbonaceous materials or residual organic compounds. At higher temperatures, both systems stabilize, reflecting the presence of thermally stable silver nanoparticles. The key difference lies in the more pronounced and phased degradation of chitosan compared to the more straightforward organic decomposition in the plant extract-based system.

### 2.3. FTIR Confirms Chemical Interaction Between Chitosan and AgNPs

**Figure 2:**
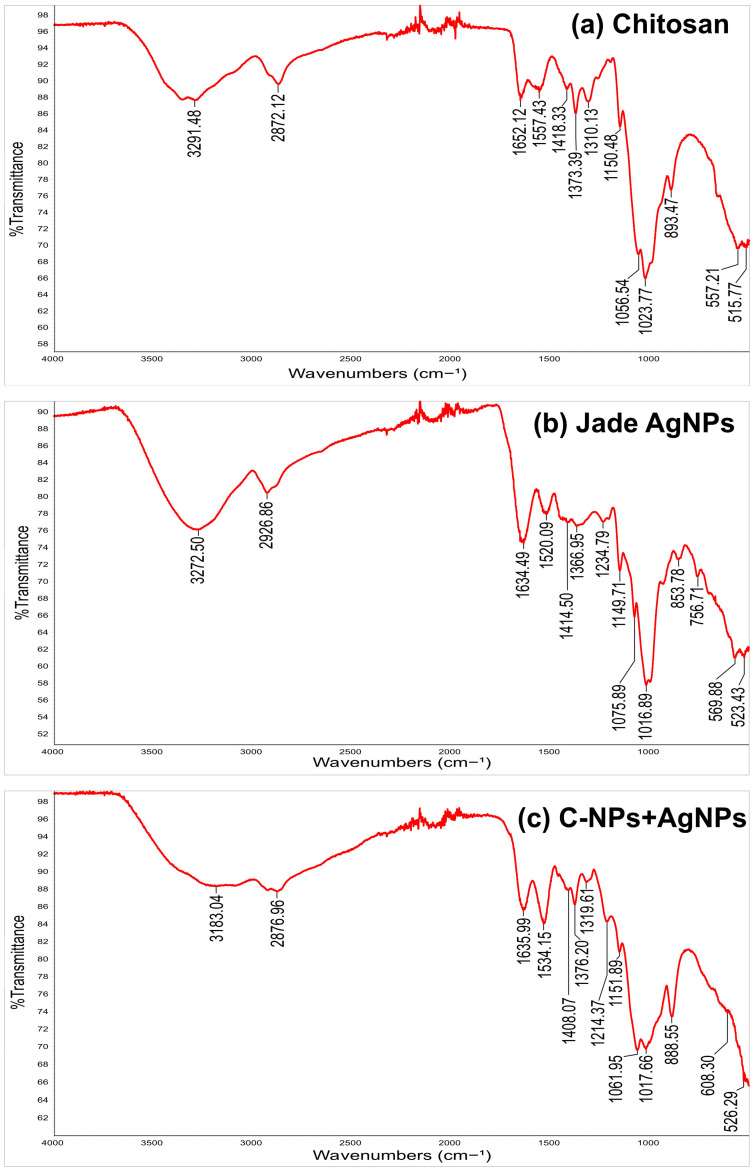
FTIR spectra of (**a**) chitosan, (**b**) Jade AgNPs, and (**c**) chitosan-coated AgNPs.

The FTIR spectra for chitosan, Jade AgNPs, and chitosan-coated AgNPs show distinct and informative peaks, reflecting the chemical interactions and structural characteristics of each sample. For chitosan, the spectrum shows a broad peak around 3291 cm^−1^, corresponding to O-H and N-H stretching vibrations, indicating the presence of hydroxyl and amine groups (Figure 2a). The peak at 2872 cm^−1^ corresponds to C-H stretching vibrations. These groups are characteristic of the polysaccharide backbone and amino groups in chitosan, confirming its natural polymeric structure. Peaks at 1652 cm^−1^ represent the amide I band (C=O stretching), while the peaks around 1150 cm^−1^ and 1056, 1023 cm^−1^ are attributed to C–O–C and C-O stretching vibrations, respectively, which are indicative of the glycosidic linkages in the chitosan structure [37].

In the spectrum for Jade AgNPs, the peak at 3272 cm^−1^ suggests O-H stretching, likely from phenolic compounds in the plant extract used for the reduction of silver ions (Figure 2b). The peak at 2926 cm^−1^ is associated with C-H stretching, likely from the aliphatic chains in plant lipids or proteins. The presence of peaks near 1634 cm^−1^ (C=O stretching) indicates organic functional groups, such as carboxyl or carbonyl, likely involved in the capping of the AgNPs. The peaks at 1149, 1075, and 1016 correspond to C-O stretching vibrations from the alcohol, glycosides, or esters present in the plant extract. These functional groups play a role in stabilizing the nanoparticles and preventing aggregation [38].

The spectrum for chitosan-coated AgNPs shows a similar peak at 3184 cm^−1^ for O-H and N-H stretching, consistent with chitosan’s structure (Figure 2c). The peak at 2876 is likely due to C-H stretching vibrations. However, the peak intensities shift slightly compared to pure chitosan and AgNPs, indicating interactions between the chitosan and the AgNP surface. A peak at 1635 cm^−1^ (C=O stretching) is observed, similar to the Jade AgNP and chitosan spectrum, indicating the presence of carboxylate and amide functional groups. The peak at 1534 cm^−1^ is due to the N-H bending in amide. Peaks near 1061 and 1017 cm^−1^ are observed, consistent with the C–O–C stretching of chitosan and the plant extract synthesized AgNPs [39]. The comparison between these three spectra shows a clear progression of functional group interactions. In the plant-synthesized AgNPs, organic compounds from the plant extract provide the capping and stabilization, as evidenced by the C=O and O-H stretching bands. After coating with chitosan, the spectrum shows both the characteristic peaks of chitosan (O-H, N-H, C–O–C) and modified intensities of the plant-based functional groups. This suggests a layered structure where chitosan encapsulates the plant-capped AgNPs, providing additional electrostatic stability.

### 2.4. SEM Reveals Effective Encapsulation and Uniform Distribution in Films

**Figure 3:**
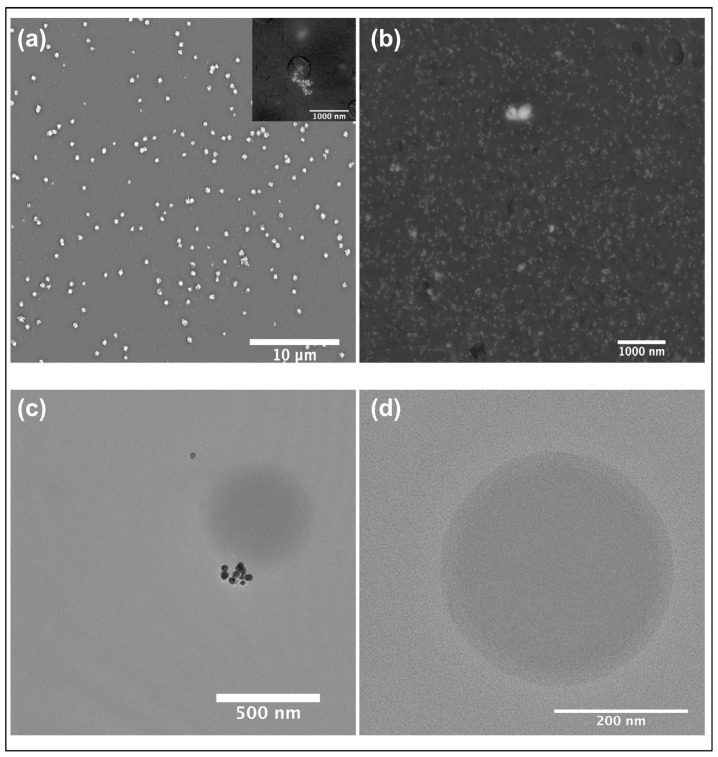
SEM micrograph of (**a**) chitosan-coated AgNPs. The inset shows a magnified image of AgNPs (bright dots) encapsulated in C-NPs (dark shell), (**b**) AgNPs-embedded in C-NPs/PVA film showing the uniform distribution of AgNPs in the polymer matrix. (**c**) TEM image of AgNPs-encapsulated in C-NPs, (**d**) bare C-NPs.

The SEM analysis of chitosan-encapsulated AgNPs was performed using a backscatter electron detector. Bright spots corresponding to the AgNPs were observed, indicating the presence of high atomic number silver within the encapsulated system (Figure 3a). Surrounding these bright spots, a darker shell corresponding to the chitosan polymer matrix was clearly visible, providing a strong contrast between the metal core and the organic coating. The average size of the encapsulated nanoparticles, including the chitosan shell, was calculated to be approximately 335 nm. Additionally, analysis of the C-NPs + AgNPs film revealed a near-uniform distribution of AgNPs within the polymer film (Figure 3b).

### 2.5. TEM Confirms Internalization of AgNPs Within Chitosan Nanoparticles

The TEM analysis of AgNPs-loaded C-NPs revealed distinct dark spots corresponding to the high atomic number of the AgNPs (Figure 3c). Surrounding these dark spots, a faint circular structure was visible, representing the chitosan matrix encapsulating the AgNPs. This contrast between the dense AgNP core and the lighter chitosan shell confirmed successful encapsulation. In comparison, the TEM images of bare C-NPs showed a faint, hollow, spherical structure, confirming the structural integrity of the polymer matrix (Figure 3d). These observations demonstrate the successful loading of AgNPs into the C-NPs and the formation of hollow spheres in bare C-NPs.

### 2.6. Mechanical Properties of Nanocomposite Films Support Potential Wound Care Applications

To assess whether the synthesized nanocomposite films possess mechanical characteristics relevant to wound care applications, dynamic mechanical analysis was performed. The results showed that incorporating AgNPs and gentamicin increased the storage modulus while decreasing the loss tangent, indicating enhanced stiffness and improved elastic performance, both desired traits for candidate wound-dressing materials.

**Figure 4:**
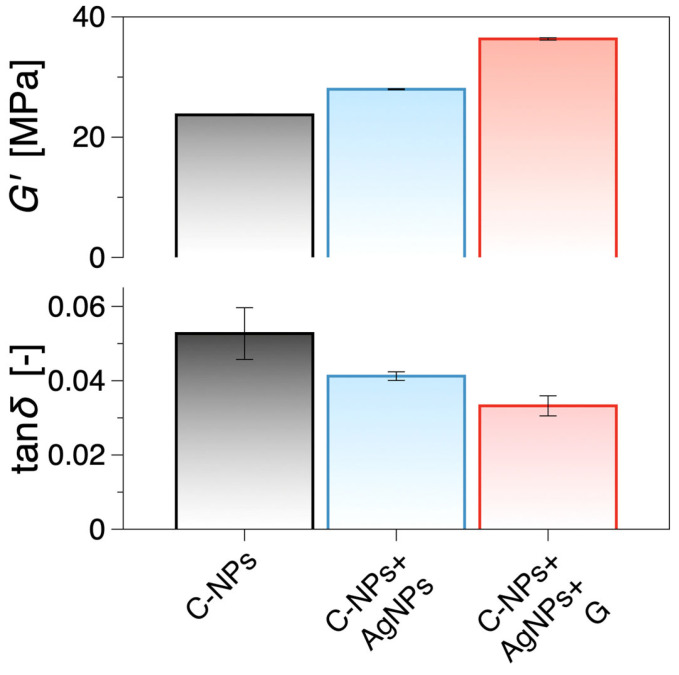
Storage shear modulus, G′, and loss tangent (or damping factor), $tan\delta =G^{\prime \prime }/G\prime$, comparing C-NPs and compositions with AgNPs and AgNPs + gentamicin (G). The data presented are the mean values in the linear viscoelastic regime from the strain sweep tests presented in Figure S1, and the error bars are the standard error of the mean. The addition of Ag nanoparticles increases G′, while the combined presence of AgNPs and gentamicin yields the highest stiffness and lowest \tan\delta. Data represent mean ± SD (n = 3).

Dynamic mechanical analysis data show that the increase in antibacterial efficacy of the films does not compromise their mechanical properties (Figure 4). On the contrary, the incorporation of AgNPs nanoparticles and gentamicin progressively increased the storage modulus and decreased the loss tangent. The reference C-NPs film displayed a storage modulus of ca. 24 MPa, which increased by ca. 16% with the addition of Ag nanoparticles and by ca. 50% to approx. 36 MPa when both AgNPs and gentamicin were incorporated. Given that the loss modulus remains largely constant across compositions (Figure S1), the decrease in loss tangent can be attributed to the increase in stiffness. This trend is consistent with mechanical reinforcement effects and restricted molecular mobility. Thus, the addition of AgNPs and gentamicin enhances the elastic response of the films without significantly altering their intrinsic dissipative behaviour.

### 2.7. Gentamicin and AgNPs Enhance Bactericidal Activity

**Figure 5:**
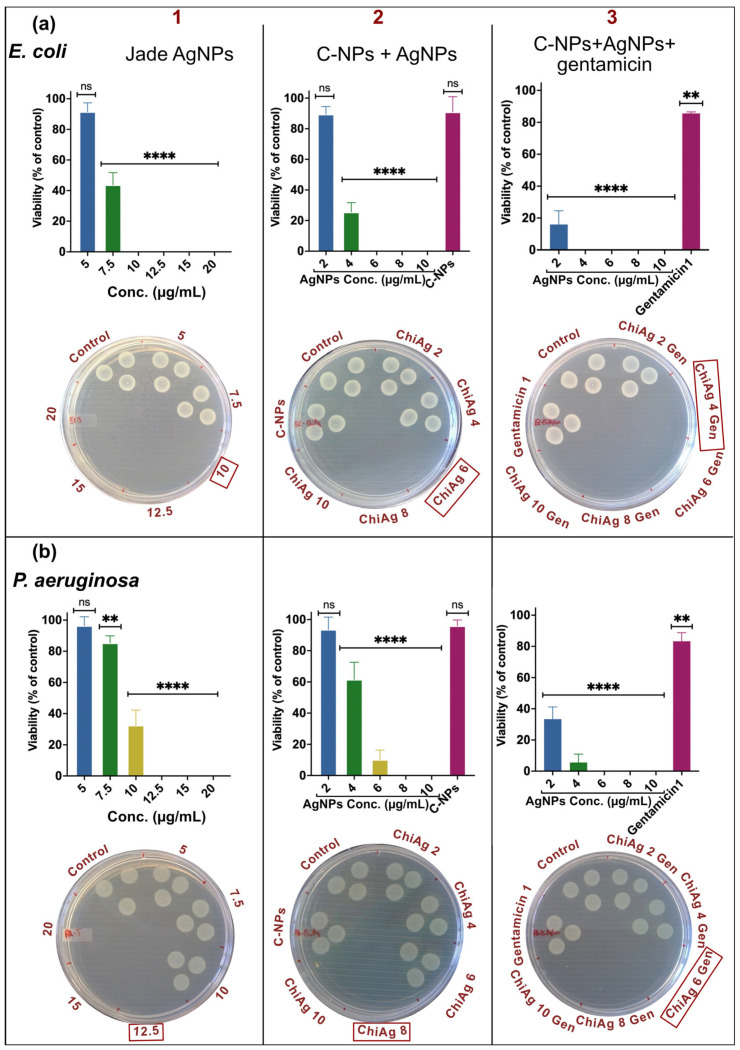
MBC of Jade AgNPs (column 1), chitosan combined with AgNPs (column 2), and chitosan with AgNPs and 1 µg/mL of gentamicin (column 3) determined using resazurin and spot assay for (**a**) *E. coli*, and (**b**) *P. aeruginosa*. Red box marks the observed MBC value. Data represent mean ± SD (*n* = 3). Statistical significance is indicated as **** *p* < 0.0001, ** *p* < 0.01, and ns = not significant.

**Figure 6:**
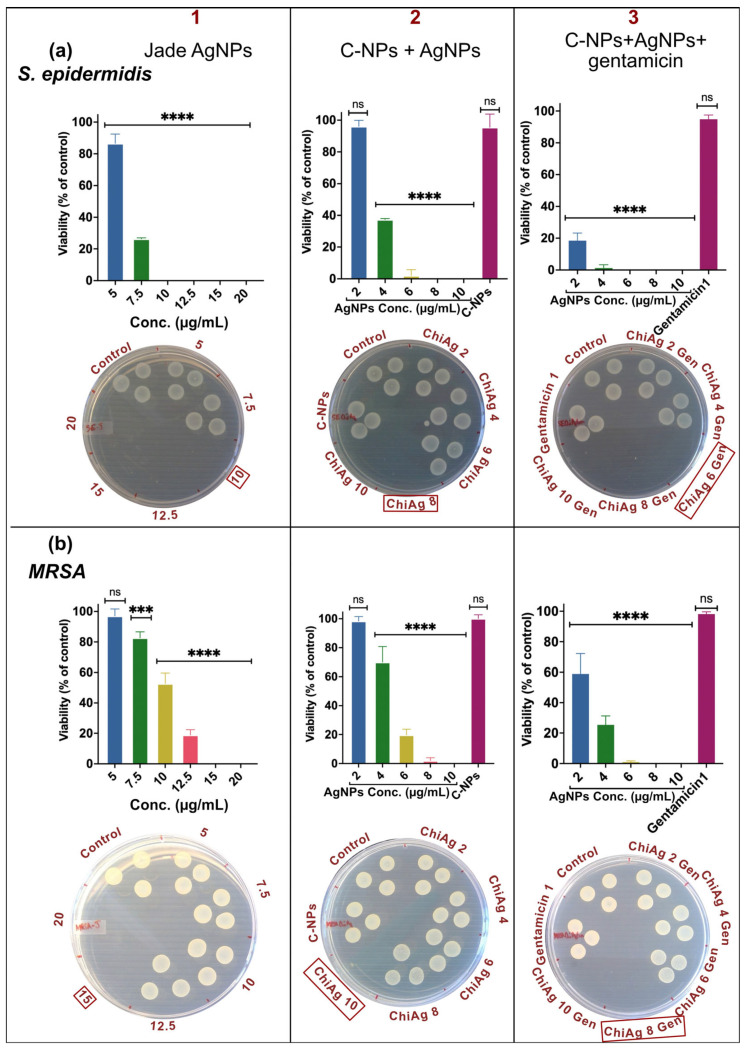
MBC of Jade AgNPs (column 1), chitosan combined with AgNPs (column 2), and chitosan with AgNPs and 1 µg/mL of gentamicin (column 3) determined using resazurin and spot assay for (**a**) *S. epidermidis*, and (**b**) MRSA. Red box marks the observed MBC value. Data represent mean ± SD (*n* = 3). Statistical significance is indicated as **** *p* < 0.0001, *** *p* < 0.001, and ns = not significant.

The MBC of AgNPs, chitosan-coated AgNPs, and chitosan-coated AgNPs loaded with 1 µg/mL gentamicin were evaluated based on fluorescent readout after incubation with alamarBlue cell viability reagent, followed by spotting 10 µL of 10× diluted samples onto MH agar plates to determine bacterial viability. Jade AgNPs alone showed a significant reduction in bacterial viability, with MBC values of 10 μg/mL for *E. coli*, 12.5 μg/mL for *P. aeruginosa*, 10 μg/mL for *S. epidermidis*, and 15 μg/mL for MRSA (Figure 5 and Figure 6 column 1). Chitosan-coated AgNPs demonstrated improvement in bactericidal efficacy at lower concentrations, with MBC values of 6 μg/mL, 8 μg/mL, 8 μg/mL, and 10 μg/mL for *E. coli*, *P. aeruginosa*, *S. epidermidis*, and MRSA, respectively, indicating the beneficial effect of the chitosan coating (Figure 5 and Figure 6 column 2). When gentamicin (1 μg/mL) was loaded along with AgNPs into the chitosan matrix, a further increase in bactericidal potency was observed, with MBC values of 4 μg/mL for *E. coli*, 6 μg/mL for *P. aeruginosa*, 6 μg/mL for *S. epidermidis*, and 8 μg/mL for MRSA (Figure 5 and Figure 6 column 3), highlighting the enhanced bactericidal activity achieved by combining AgNPs with chitosan and gentamicin. Overall, the results show that while AgNPs are effective at higher concentrations, chitosan coating and gentamicin loading significantly improve bactericidal efficiency at much lower concentrations.

**Figure 7:**
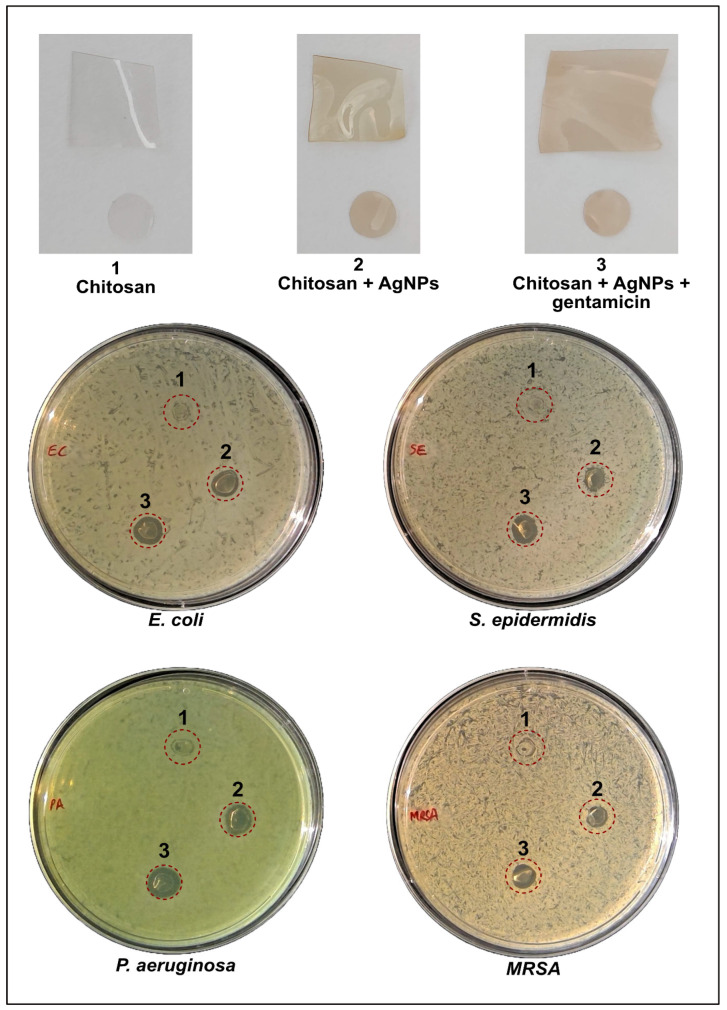
Polymer films of (**1**) bare chitosan/PVA, (**2**) chitosan/PVA + AgNPs, and (**3**) chitosan/PVA + AgNPs + gentamicin. The agar plates show the disc diffusion test of the three types of polymer films on *E. coli*, *P. aeruginosa*, *S. epidermidis*, and MRSA.

In the disc diffusion test, bare chitosan film showed no observable inhibition against bacterial growth (Figure 7). However, when AgNPs were loaded into the chitosan film, a clear zone of inhibition was observed, indicating the AgNPs’ bactericidal effect (Table 1). The addition of 1 µg/mL gentamicin to the AgNPs-loaded chitosan film further enhanced the antibacterial activity, resulting in a more pronounced zone of inhibition, demonstrating an enhanced antibacterial effect when AgNPs and gentamicin are combined within the chitosan matrix.

## 3. Discussion

In this study, we investigated the antimicrobial potential of AgNPs synthesized from *C. ovata* leaf extract and their enhancement through combination with chitosan and gentamicin. The results confirmed that combining AgNPs with chitosan significantly improved the antimicrobial efficacy of the nanoparticles at lower concentrations. Chitosan’s ability to form a protective layer around AgNPs not only increased their stability but also facilitated better interaction with bacterial cells [40]. This effect can be attributed to the strong electrostatic interactions between the positively charged amino groups in chitosan and the negatively charged bacterial membranes, leading to greater attachment and penetration of the nanoparticles, as suggested in other studies where chitosan was used to enhance the bactericidal activity of AgNPs [41]. Beyond antimicrobial performance, our dynamic mechanical analysis provided valuable insights into the structural reinforcement achieved through AgNPs and gentamicin incorporation. The observed increase in the storage modulus (G′) and the corresponding decrease in loss tangent (tan δ) indicate enhanced stiffness and reduced molecular mobility within the chitosan film matrix. This mechanical improvement implies stronger intermolecular interactions and better dispersion of AgNPs and gentamicin within the polymeric network, enhancing the film’s integrity and resistance to deformation.

Previous studies on polymer-based wound dressings emphasize that mechanical robustness, elasticity, and structural integrity are essential for durability, conformability, and comfortable handling. In this context, the storage modulus of our films increased from approximately 24 MPa in the chitosan control to ~36 MPa in the AgNPs-gentamicin composite, placing them within a desirable range for flexible yet durable wound dressing materials. Comparable studies on chitosan-based films incorporating metallic nanoparticles have reported similar improvements in stiffness and reductions in damping behaviour, consistent with the trends observed here [42,43,44]. These enhanced mechanical characteristics, together with the strong antibacterial performance of the nanocomposite, highlight its potential to function as a robust wound dressing material capable of providing both structural integrity and effective infection control.

The chitosan-coated AgNPs exhibited lower MBC values compared to AgNPs alone, with MBC values of 6 µg/mL for *E. coli* and 8 µg/mL for *P. aeruginosa*, *S. epidermidis*, and 10 µg/mL for MRSA. These results are consistent with findings from similar studies where the combination of chitosan and AgNPs was shown to improve antimicrobial efficacy. For instance, Elmehbad et al. demonstrated that chitosan-AgNP nanocomposites exhibited enhanced antimicrobial activity against various bacterial strains due to the combined effects of chitosan and AgNPs, reducing the required bactericidal doses [42]. Furthermore, Sathiyaseelan et al. reported that the chitosan matrix not only stabilized the nanoparticles but also improved their biocompatibility, further supporting the results observed in our study [43].

The incorporation of 1 µg/mL gentamicin into the chitosan-AgNPs matrix further amplified the bactericidal activity, with MBC values dropping to 4 µg/mL for *E. coli*, 6 µg/mL for *P. aeruginosa*, *S. epidermidis*, and 8 µg/mL for MRSA. This result highlights the enhanced combined antibacterial effect between AgNPs, chitosan, and gentamicin, where the nanoparticles disrupt bacterial membranes while gentamicin interferes with bacterial protein synthesis, thus enhancing the overall antimicrobial effect. While the observed reduction in MBC values upon gentamicin incorporation indicates enhanced combined antibacterial activity, formal synergy quantification (e.g., checkerboard assays to determine fractional inhibitory concentration indices or time-kill kinetics) will be required in future work to distinguish additive versus synergistic interactions.

This observation aligns with other studies showing that the combination of AgNPs with antibiotics can potentiate the antimicrobial effects, particularly against multidrug-resistant bacteria [45,46]. Similar synergistic effects were demonstrated by Smekalova et al., who found that AgNPs combined with various antibiotics, especially gentamicin, significantly reduced bacterial viability at lower concentrations compared to either agent alone [47]. Additionally, Malawong et al. also studied the antimicrobial efficacy of AgNPs-antibiotic combinations and found that the synergism between antibiotics and AgNPs resulted in a multi-fold increase in antimicrobial efficacy [48].

The bactericidal performance observed for the gentamicin-loaded composite films compares favourably with previously reported chitosan-AgNPs systems, where strong antibacterial activity has been widely demonstrated in film form [41,49,50]. The observed reduction in MBC values upon gentamicin incorporation is consistent with reports that antibiotic loading into chitosan-based films and nanocomposites can enhance antimicrobial potency while supporting localized infection-control concepts [51,52]. Similarly, the measured increase in storage modulus (≈24 MPa to ≈36 MPa) aligns with reports of improved dynamic mechanical performance in chitosan films doped with silver nanoparticles and comparable antimicrobial nanocomposite film systems, supporting improved robustness without compromising elasticity [53,54,55].

In addition to the broth microdilution assays, the disc diffusion test provided further validation of the enhanced antibacterial properties of the multifunctional nanocomposites. Bare chitosan films alone showed no observable inhibition against bacterial growth, consistent with previous reports that while chitosan possesses inherent antimicrobial properties, its effect is limited without the presence of nanoparticles. In contrast, the AgNPs-loaded chitosan films exhibited clear zones of inhibition, demonstrating the effective bactericidal action of the AgNPs. Similar results were observed by Venkatesan et al., who reported that while chitosan-alginate composite films did not show any antimicrobial activity, their combination with AgNPs greatly enhanced the antimicrobial efficacy of the nanocomposite [56]. Moreover, the addition of gentamicin to the AgNPs-chitosan films further enhanced the antibacterial activity, resulting in a more pronounced zone of inhibition. This outcome is in line with findings from Yu et al., where the inclusion of antibiotics in nanoparticle-loaded films resulted in a broader and more effective antibacterial response [57]. It is important to note that the limited diffusion of both AgNPs and gentamicin from the chitosan-based films may be advantageous in controlled release applications. This restricted diffusion may help localize the antimicrobial agents directly at the site of infection, which is particularly beneficial for applications such as wound healing, where sustained and targeted antimicrobial activity is crucial. The slow release of AgNPs and gentamicin ensures prolonged exposure to the pathogens, reducing the risk of infection recurrence and minimizing the need for frequent reapplication of the treatment. Additionally, the site-specific release minimizes systemic exposure to antibiotics, which can help mitigate potential side effects and reduce the likelihood of developing antibiotic resistance; however, release kinetics were not quantified in the present study and should be evaluated in future work.

The ability of chitosan films to control the diffusion of bioactive compounds makes them an ideal platform for localized therapeutic applications. However, quantitative release measurements will be necessary to validate these effects [33]. The antimicrobial activity observed for the composite films is likely influenced by both nanoparticle–bacteria interactions at the film surface and diffusion of soluble antimicrobial species (e.g., Ag^+^ and gentamicin) from the polymer matrix. However, release kinetics and diffusion behaviour were not quantified in the present study. Systematic profiling of gentamicin and Ag^+^ release under wound-relevant conditions (including pH and ionic strength) will therefore be essential to evaluate localized versus sustained antimicrobial activity and to optimize dosing for translational applications.

In conclusion, our findings underscore the significant advantages of combining AgNPs with chitosan and gentamicin to create a multifunctional nanocomposite with enhanced antibacterial properties. The chitosan coating not only improves the stability and delivery of AgNPs but also contributes to their antibacterial efficacy by promoting interactions with bacterial cell walls. The incorporation of gentamicin further enhances the antimicrobial potential of the composite, making it a promising candidate for further evaluation in applications such as wound healing, where frequent infections due to bacterial resistance are a major concern [58]. The ability of this nanocomposite to lower the required bactericidal concentrations while maintaining high efficacy also suggests its potential for use in treatments where minimizing antibiotic dosage is critical to reducing side effects and preventing the emergence of resistant bacterial strains [59,60].

***Biocompatibility considerations:*** It is well recognized that the cytotoxicity of AgNPs-containing materials depends strongly on nanoparticle dose, surface chemistry, and exposure time, and therefore cytocompatibility evaluation is essential prior to biomedical translation [61,62]. Previous studies have reported that chitosan-based matrices can reduce AgNPs-associated cytotoxicity by limiting direct nanoparticle exposure and moderating ion release [63,64]. Additionally, plant-derived surface capping agents may further influence biocompatibility [65,66]. Standardized cytotoxicity testing (e.g., extract-based and direct-contact methods) is therefore required to define safe concentration windows for wound healing applications.

***Limitations and future work:*** Although the films demonstrate strong antimicrobial activity and improved mechanical performance, further studies evaluating cytocompatibility, hemocompatibility, release kinetics, and *in vivo* wound healing efficacy are required prior to biomedical translation. In addition, while DLS and zeta potential measurements support the colloidal stability of the synthesized nanoparticles under the tested conditions, the present study did not include a systematic stability assessment across storage time, temperature, and pH. Such profiling will be critical to evaluate formulation robustness under physiological media and wound-relevant pH environments. Future work will therefore include time-resolved DLS monitoring under different pH and temperature conditions, alongside release profiling and quantitative cytocompatibility and hemocompatibility testing.

## 4. Materials and Methods

Silver nitrate (AgNO_3_), tryptic soy broth (TSB), Mueller Hinton (MH) medium, Dulbecco’s phosphate-buffered saline (DPBS), acetic acid, chitosan (low molecular weight), and poly(vinyl alcohol) were purchased from Sigma Aldrich (Merck), Søborg, Denmark. Sodium tripolyphosphate (STPP) and alamarBlue™ HS Cell Viability Reagent were purchased from Thermo Fisher Scientific, Roskilde, Denmark.

### 4.1. AgNP Synthesis

*C. ovata* leaf extract was used for the biological synthesis of AgNPs, similar to a previously reported process with minor modifications [67]. The process involved washing and cutting the leaves, adding 25 g to a sterile flask with 100 mL of distilled water, autoclaving at 121 °C for 15 min, and filtering the resulting phytochemical extract. To this extract, various concentrations of AgNO_3_ were added, and the resulting solution was stirred at room temperature (RT). The change in colour of the solution to dark brown and UV-Vis spectral analysis confirmed AgNPs synthesis. Post synthesis, the AgNPs were washed by initial centrifugation at 5000× *g* for 3 min to remove larger particles, followed by three washes at 45,000× *g* with distilled water to eliminate unreacted silver salt and leaf extract. The resulting pellet was reconstituted in Milli-Q water and stored in a refrigerator.

### 4.2. Chitosan Nanoparticle Synthesis

Chitosan nanoparticles (C-NPs) were synthesized using a previously reported procedure with slight modifications [68]. Briefly, 1% chitosan solution was prepared by adding chitosan to 1% acetic acid solution to achieve a final concentration of 10 mg/mL. The solution was stirred overnight to dissolve the chitosan completely. For nanoparticle synthesis, the stock chitosan solution was diluted to 0.05% with Milli-Q water, and the pH of the solution was adjusted to 4.5 with 1M NaOH solution. To this chitosan solution, a 0.05% solution of STPP was added in a 1:3 ratio, STPP: chitosan, and the mixture was homogenized at 6.5 k RPM for 1 min using a Precellys^®^ 24 homogenizer (Bertin Technologies, Montigny-le-Bretonneux, France). The resulting nanoparticles were washed by centrifugation at 17,000× *g* for 20 min (3×) with distilled water.

For the synthesis of AgNPs-loaded C-NPs (C-NPs + AgNPs), after dilution of the stock chitosan solution and adjusting the pH, AgNPs were added to the desired concentration, mixed well using a vortex shaker, and followed by similar steps as those of C-NPs synthesis. Similarly, for gentamicin loading, the desired concentration of gentamicin was added along with AgNPs, mixed by vortexing, followed by the addition of STPP and homogenization.

For producing chitosan films, 0.05% C-NPs, C-NPs with AgNPs, and C-NPs + AgNPs + gentamicin (1 µg/mL) were mixed with 1.5% PVA solution to act as a plasticizer, drop-cast into a dish, and allowed to air dry.

For clarity, formulations are referred to as follows: chitosan nanoparticles (C-NPs), silver nanoparticles synthesized using Jade leaf extract (Jade AgNPs), and gentamicin-loaded composite films (C-NPs + AgNPs + Gen). Films without gentamicin are referred to as C-NPs + AgNPs.

### 4.3. Characterization of Nanoparticles

#### 4.3.1. Dynamic Light Scattering (DLS)

For DLS analysis, nanoparticle suspensions were diluted with Milli-Q water to obtain the optimal concentration for accurate DLS analysis. A 1 mL aliquot of the diluted suspension was examined using a Malvern Zetasizer Nano ZS90 instrument (Malvern Panalytical, Malvern, UK), which provided measurements of the nanoparticles’ hydrodynamic diameter and polydispersity index (PDI). Additionally, the zeta potential was determined using a folded capillary zeta cell in order to assess the stability of the nanoparticles. These parameters were used as indicators of dispersion stability and aggregation propensity under the measurement conditions. A comprehensive stability analysis across extended storage times and varying pH/temperature conditions was not performed in this study and is included as future work.

#### 4.3.2. Fourier Transform-Infrared Spectroscopy (FTIR)

FTIR spectroscopy was employed to identify the functional groups involved in the synthesis of AgNPs loaded into C-NPs. The AgNPs-loaded chitosan samples were dried before analysis. FTIR spectra were recorded using a Nicolet iS50 spectrometer (Thermo Fisher Scientific, Waltham, MA, USA) in the wavelength range of 4000–500 cm^−1^ at a resolution of 4 cm^−1^. The spectrum of pure chitosan and AgNPs was also collected for comparison.

#### 4.3.3. Thermogravimetric Analysis (TGA)

The thermal stability of AgNPs and C-NPs + AgNPs was assessed using TGA on a Discovery TGA, TA Instrument. The samples were heated from ambient temperature to 900 °C with a ramp rate of 10 °C/min.

#### 4.3.4. Scanning Electron Microscopy (SEM)

SEM was used to visualize bare C-NPs and AgNP-encapsulated C-NPs. The samples were diluted, and a drop was cast onto a diced silicon wafer affixed on an aluminum stub using an adhesive carbon tab. The samples were imaged using a Quanta FEG-250 SEM (FEI Company, Hillsboro, OR, USA) equipped with a backscattered electron (BSE) detector.

#### 4.3.5. Transmission Electron Microscopy (TEM)

C-NPs and AgNP-encapsulated C-NPs were examined using TEM. Diluted nanoparticle suspensions were placed on a carbon-coated copper grid and air-dried at RT. TEM analysis was carried out to obtain details about the size, shape, and distribution of the nanoparticles using a FEI Tecnai T20 G2 (FEI Company, Hillsboro, OR, USA) operated at an accelerating voltage of 200 kV.

### 4.4. Mechanical Testing of Films

The mechanical properties of the fabricated films were assessed using dynamic mechanical analysis (DMA) in torsion. In torsional linear viscoelastic DMA testing, samples are subjected to a sinusoidal shear deformation with a strain amplitude and frequency, with the stress response delayed by a phase angle due to material relaxation. Thus, DMA is a non-destructive testing method capable of assessing the viscoelastic material response of soft materials. By separating the stress output into an in-phase (relative to the strain input) and an out-of-phase component, the mechanical response is thus characterized by a shear storage (elastic; in-phase) modulus and a shear loss (viscous; out-of-phase) modulus. The tests were performed on an Anton Paar MCR702e Space (Graz, Austria) rotational rheometer using a torsional rectangular fixture. All tests were performed at room temperature (23 °C) and relative humidity (approx. 40%). The test sample dimensions were 0 mm, with the thickness varying between 30 and 50 µm. DMA measurements were performed on three independently prepared films for each formulation (*n* = 3). The linear viscoelastic strain amplitude limit was determined from strain amplitude sweep tests, %, and constant frequency, Hz. Here we report the mean and standard error of the mean within the linear viscoelastic region (LVE—strain amplitude region where the moduli are largely strain-amplitude independent) of the storage modulus, and the corresponding loss tangent (or damping factor) as a measure of their viscoelastic character. The strain-sweep data are shown in Figure S1. Complementarily, linear viscoelastic frequency-sweep tests were also performed to cross-check the strain-sweep data (Figure S2).

### 4.5. Antibacterial Activity Evaluation

#### 4.5.1. Bacterial Strains

The antimicrobial activity was evaluated against four pathogens, comprising two Gram-positive bacteria (*Staphylococcus epidermidis* ATCC 35984 and Methicillin-resistant *Staphylococcus aureus* (MRSA) USA300_FPR3757) and two Gram-negative bacteria (*Escherichia coli* UTI 89 and *Pseudomonas aeruginosa* PAO1). *E. coli* and *P. aeruginosa* were grown in LB medium, and TSB was used for *S. epidermidis* and *S. aureus* growth. Antimicrobial studies were conducted in MH medium.

#### 4.5.2. Minimum Bactericidal Concentration (MBC)

The broth microdilution method was used to determine the MBC of nanoparticle combinations. Overnight cultures of bacterial strains were diluted to approximately 5 × 10^5^ CFU/mL in MH medium. Varying concentrations of AgNPs, C-NPs + AgNPs, and C-NPs + AgNPs + gentamicin were added to a 96-well microtiter plate. Each well was inoculated with diluted bacterial culture and incubated at 37 °C for 20 h. After overnight incubation, 10× alamarBlue HS cell viability reagent was added to the wells and incubated for 1 h at 37 °C. The fluorescence was measured using an excitation/emission wavelength of 560/590 nm. Furthermore, the samples were diluted ten times, and 10 µL were spotted on an MH agar plate and incubated overnight. The MBC was determined as the lowest concentration, which resulted in a 99.9% reduction in the initial bacterial count, demonstrating a bactericidal effect. MBC measurements were performed in triplicate (*n* = 3) for each pathogen.

Additionally, to test whether there was any change in antimicrobial efficacy after film formation or due to the addition of PVA as a plasticizer, a disc diffusion test was conducted. Briefly, after film formation, C-NPs, C-NPs + AgNPs, and C-NPs + AgNPs + gentamicin films were cut into uniform discs using a 5 mm biopsy punch. Overnight bacterial cultures were diluted to an OD of 0.08, 100 µL was spread onto MH agar plates, and the discs were added. After overnight incubation, the plates were assessed for the zone of inhibition.

### 4.6. Statistical Analysis

All experiments were performed in at least three independent replicates unless otherwise stated. Results are presented as mean ± standard deviation (SD). Statistical significance was evaluated using one-way ANOVA followed by Tukey’s post hoc test (GraphPad Prism, version 10.6.1), with *p* < 0.05 considered significant.

## 5. Conclusions

This study demonstrated the significant advantages of combining Jade-plant-derived AgNPs with chitosan and gentamicin to create a multifunctional nanocomposite with enhanced antibacterial properties. The combined effects of these components resulted in improved bactericidal efficacy at lower concentrations, as shown through the MBC and disc diffusion assays. Chitosan played a crucial role in stabilizing the AgNPs, facilitating better interactions with bacterial cell membranes, and providing a polymer matrix that may support a sustained-release platform for gentamicin. The incorporation of gentamicin further broadened the antimicrobial spectrum of the nanocomposite, offering potent activity against both Gram-positive and Gram-negative bacteria.

In addition, dynamic mechanical analysis revealed that embedding AgNPs and gentamicin increased the storage modulus and reduced the loss tangent of the films, signifying improved stiffness and elasticity. Importantly, the films exhibited mechanical performance within a range reported in the literature for polymer films investigated for wound dressing applications, supporting further evaluation. This indicates that the nanocomposite not only displays strong antibacterial activity but also possesses the structural integrity and durability required for applications such as wound dressings, coatings, and drug delivery films.

Taken together, these findings highlight the potential of this multifunctional nanocomposite in biomedical applications, particularly in wound healing and localized drug delivery, where sustained antimicrobial action must be complemented by reliable mechanical strength.

## Figures and Tables

**Table 1:** Observed zone of inhibition after incubation with the polymer discs.

Bacteria	Chitosan (mm)	Chitosan + AgNPs (mm)	Chitosan + AgNPs + Gentamicin (mm)	% Increase Over Chi-AgNPs
*E. coli*	0	6.76	8.07	19.38
*P. aeruginosa*	0	7.15	8.8	23.08
*S. epidermidis*	0	5.58	7.67	37.46
MRSA	0	5.6	6.87	22.68

## Data Availability

The original contributions presented in this study are included in the article/Supplementary Materials. Further inquiries can be directed to the corresponding author(s).

## Supplementary Materials

The supporting information can be downloaded at https://www.mdpi.com/article/10.3390/ijms27021036/s1.
