# Supplementary material for: A Novel Approach to Combating Antibiotic Resistance: A Chitosan-Based Nanocomposite with Green AgNPs and Gentamicin
Source: Int J Mol Sci. 2026 Jan 20;27(2):1036. doi: 10.3390/ijms27021036 (PMC12842585; doi:10.3390/ijms27021036)
Supplement: Supplementary file 1 [file ijms-27-01036-s001.zip › ijms-4083826-supplementary.pdf]

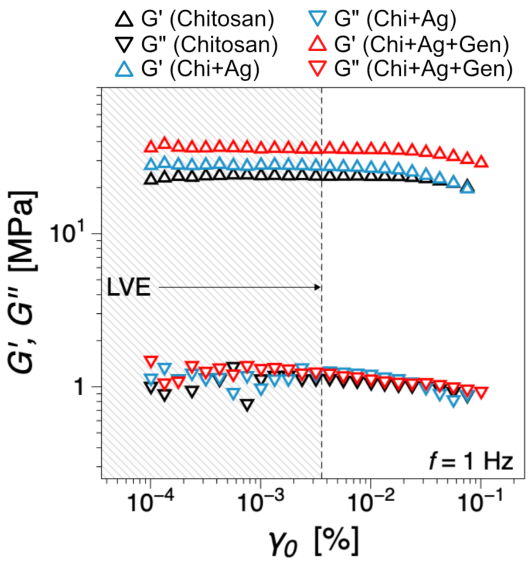

Figure S1. Shear storage and loss moduli with increasing strain amplitude in a strain sweep test. LVE refers to the approximate limit of the Linear Viscoelastic region.

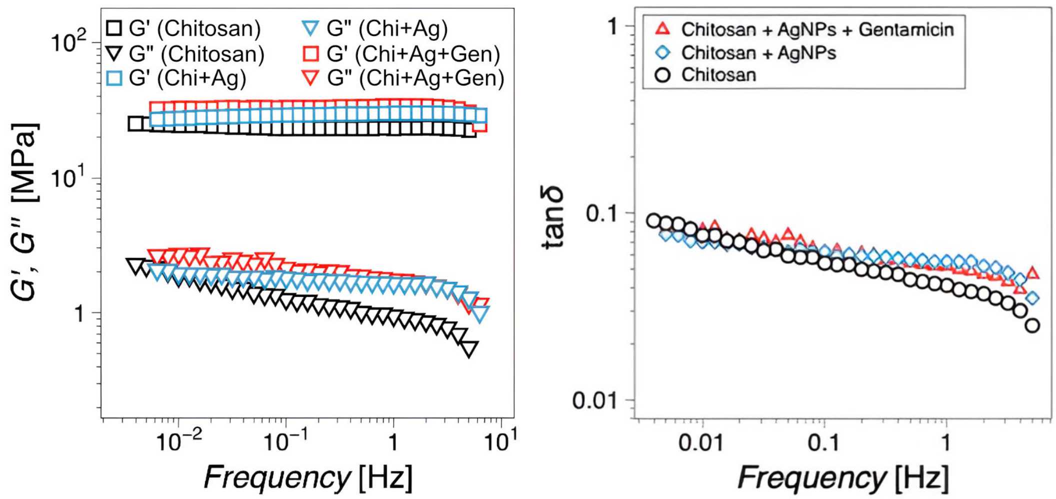

Figure S2. Dynamic moduli (storage and loss) during frequency sweep tests and the corresponding loss factor.
